# Post-treatment With Qing-Ying-Tang, a Compound Chinese Medicine Relives Lipopolysaccharide-Induced Cerebral Microcirculation Disturbance in Mice

**DOI:** 10.3389/fphys.2019.01320

**Published:** 2019-10-25

**Authors:** Hao-Min Wang, Ping Huang, Quan Li, Lu-Lu Yan, Kai Sun, Li Yan, Chun-Shui Pan, Xiao-Hong Wei, Yu-Ying Liu, Bai-He Hu, Chuan-She Wang, Jing-Yu Fan, Jing-Yan Han

**Affiliations:** ^1^Department of Integration of Chinese and Western Medicine, School of Basic Medical Sciences, Peking University, Beijing, China; ^2^Tasly Microcirculation Research Center, Peking University Health Science Center, Beijing, China; ^3^Key Laboratory of Microcirculation, State Administration of Traditional Chinese Medicine of the People’s Republic of China, Beijing, China; ^4^Key Laboratory of Stasis and Phlegm, State Administration of Traditional Chinese Medicine of the People’s Republic of China, Beijing, China; ^5^State Key Laboratory of Core Technology in Innovative Chinese Medicine, Beijing, China; ^6^Beijing Microvascular Institute of Integration of Chinese and Western Medicine, Beijing, China

**Keywords:** leukocyte, hyperpermeability, NF-κB, Src, tight junctions, caveolin-1

## Abstract

**Objective**: Lipopolysaccharide (LPS) causes microvascular dysfunction, which is a key episode in the pathogenesis of endotoxemia. This work aimed to investigate the effect of Qing-Ying-Tang (QYT), a compound Chinese medicine in cerebral microcirculation disturbance and brain damage induced by LPS.

**Methods**: Male C57/BL6 mice were continuously transfused with LPS (7.5 mg/kg/h) through the left femoral vein for 2 h. QYT (14.3 g/kg) was given orally 2 h after LPS administration. The dynamics of cerebral microcirculation were evaluated by intravital microscopy. Brain tissue edema was assessed by brain water content and Evans Blue leakage. Cytokines in plasma and brain were evaluated by flow cytometry. Confocal microscopy and Western blot were applied to detect the expression of junction and adhesion proteins, and signaling proteins concerned in mouse brain tissue.

**Results**: Post-treatment with QYT significantly ameliorated LPS-induced leukocyte adhesion to microvascular wall and albumin leakage from cerebral venules and brain tissue edema, attenuated the increase of MCP-1, MIP-1α, IL-1α, IL-6, and VCAM-1 in brain tissue and the activation of NF-κB and expression of MMP-9 in brain. QYT ameliorated the downregulation of claudin-5, occludin, JAM-1, ZO-1, collagen IV as well as the expression and phosphorylation of VE-cadherin in mouse brain.

**Conclusions**: This study demonstrated that QYT protected cerebral microvascular barrier from disruption after LPS by acting on the transcellular pathway mediated by caveolae and paracellular pathway mediated by junction proteins. This result suggests QYT as a potential strategy to deal with endotoxemia.

## Introduction

Lipopolysaccharide (LPS) causes endotoxemia and sepsis, leading to multiple organ failure and infectious shock resulting in a range of disastrous sequels (Berger et al., 2017). Encephalopathy is a common manifestation of endotoxemia (Siami et al., 2008), with a mortality rate of 76.1%, of which the prognosis is mostly neurological dysfunction or persistent vegetative state (Tong et al., 2015). Despite application of fluid resuscitation, vasoactive medicines and glucocorticoid, dealing with endotoxemia remains a challenge for clinician.

LPS binds to toll like receptor 4 (TLR-4) (Tsukamoto et al., 2018) and activates nuclear transcription factor κB (NF-κB), causing the release of inflammatory factors and the expression of chemotactic molecules and adhesion molecules, promoting the recruitment of leukocytes in venules (Bienvenu and Granger, 1993; Khakpour et al., 2015), resulting in microvascular occlusion and tissue ischemia (Cohen, 2002). Reduced tissue perfusion stimulates the conversion of xanthine dehydrogenase to xanthine oxidase, which brings about a massive surge of free radicals, including superoxide, hydrogen peroxide, and hydroxyl radical (Jean-Baptiste, 2007). Free radicals can activate matrix metalloproteinases (MMPs) (Vanlaere and Libert, 2009) and induce the degradations of tight junctions (TJs), leading to brain blood barrier (BBB) breakdown (Coisne and Engelhardt, 2011). As a major component of BBB, endothelial permeability is regulated by transcellular pathway mediated by the caveolae and paracellular pathway mediated by endothelial cell junctions (Keaney and Campbell, 2015). LPS recognized by TLR4 activates Src family protein tyrosine kinases (Plociennikowska et al., 2015), which in turn induces NF-κB translocation or directly activates Rock kinase and myosin light chain kinase (MLCK) signaling that causes disruption of endothelial cell junctions (Clark et al., 2015) and impairs integrity of vascular basement membrane, resulting in hyperpermeability, hemorrhage and thrombosis (Mombouli and Vanhoutte, 1999), which eventually lead to multiple organ failure, septic shock and even death (Khakpour et al., 2015). Therefore, ameliorating microvascular hyperpermeability and edema is anticipated as an important treatment strategy.

Qing-Ying-Tang (QYT) is a traditional Chinese medicine formula consisting of nine different decoction pieces. Currently, QYT has been used in clinic in China to cope with various disorders, ranging from dermatological diseases to acute infectious diseases including endotoxemia (Zhang et al., 2009; Gao et al., 2014). Clinical trials have shown that QYT combined with Western medicine for the treatment of sepsis can not only quickly eliminate the clinical symptoms and decrease the occurrence of multiple organ dysfunction syndromes, but also reduce the incidence of complications (Ji et al., 2015). In addition, some other beneficial role of QYT has been reported in clinic, such as regulation of blood fat and humoral immunity in patients with thromboangiitis obliterans (Fu, 2005), curative effect for viral encephalitis patients, who are not sensitive to hormones and anti-inflammatory drugs (Dong, 2007), reduction of high fever after abdominal surgery (Feng and Xu, 2015). However, only limited study is available so far, mostly in Chinese, regarding the rationale behind the effect of QYT on endotoxemia. On the other hand, we have previously shown that catalpol, an ingredient of the QYT formula, can reduce LPS-caused mesenteric microvascular hyperpermeability and hemorrhage (Zhang et al., 2016). DLA (Li et al., 2012) and salvianolic acid B (Lin et al., 2013), the other two constituents of QYT, have been reported to improve LPS-induced mouse cerebral microcirculation and rat lung microcirculation disorders, respectively. In view of the findings above, we speculated that the effect of QYT on endotoxemia may implicate improvement of microvascular hyperpermeability. This study was designed to test this speculation.

## Materials and Methods

### Animals

The animals used in the present study were C57/BL6 mice, male, with body weight of 18–22 g, which were purchased from Beijing Vital River Laboratory Animal Technology Company Limited (certificate no SCKX 2016-0011), and kept in an environment of temperature of 23 ± 2°C and relative humidity of 40 ± 5% with a 12-h light/dark cycle. The animals were fed with rodent food and water but underwent fasted for 12 h before experiment with allowing for access to water ad libitum. The experimental procedures were carried out as per the European commission guideline (2010/63/EU). All animals were handled based on the guidelines of the Peking University Animal Research Committee. The Committee on the Ethics of Animal Experiments of Peking University Health Science Center approved the surgical procedures and experimental protocol (LA2017-183).

### Reagents

QYT granules were obtained from Guangdong Yi Fang Pharmaceutical Co Ltd. (Guangzhou, GD, China), which consist of *rhinoceros horn* (xijiao) [*buffalo horn* (shuiniujiao) instead] (32.7%), *rehmannia glutinosa* (shengdihuang) (6.4%), *scrophularia* (yuanshen) (9.8%), *bamboo leaf* (zhuyexin) (3.2%), *Ophiopogon japonicus* (maidong) (9.7%), *salvia miltiorrhiza* (danshen) (6.5%), *coptis* (huanglian) (5.4%), *honeysuckle* (jinyinhua) (9.8%), and *forsythia* (lianqiao) (6.5%). LPS, fluorescein isothiocynate (FITC)-conjugated bovine serum albumin (FITC-BSA), Cresyl violet acetate and Evans blue were from Sigma Chemical (St. Louis, MO, United States). Rhodamine 6G was purchased from Fluka Chemie AG (Buchs, Switzerland). Antibodies against occludin, JAM-1, ZO-1, cav-1, phosphor-cav-1, VE-cadherin, and GAPDH were obtained from Cell Signaling Technology (Beverly, MA, United States). Assay kit for cathepsin B and antibody against claudin-5 were purchased from Invitrogen Corporation (Camarillo, CA, United States). Antibodies against VCAM-1, NF-κB p65, phosphor-p65, p50, and TLR-4 were obtained from Santa Cruz Biotechnology (SantaCruz, CA, United States). Antibodies against ICAM-1, Src, phosphor-Src, CD18, CD68, Iba1, collagen IV, and MMP-9 were obtained from Abcam (Cambridge, United Kingdom).

### Animal Grouping for Experiment

Five groups were set up in this study: (1) NS group, (2) NS + QYT group, (3) LPS 4 h group, (4) LPS 24 h group, and (5) LPS + QYT group, 3 9 mice in each (see Table 1 for detail). Animals were anesthetized using 2% pentobarbital sodium (60 mg/kg body weight, i.p.), and treated as follows. The mice in LPS 4 h group, LPS 24 h group and LPS + QYT group received an uninterrupted infusion of LPS solution in saline (7.5 mg/kg/h) for 2 h through left femoral vein, meanwhile, the animals in NS group and NS + QYT group received the same amount of vehicle the same way. Upon awaking from anesthesia, the mice were permitted to eat freely. Four hours thereafter, the mice in NS + QYT group and LPS + QYT group were orally administered with QYT (14.3 g/kg), while those in NS group, LPS 4 h group and LPS 24 h group received equal amount of NS in the same manner. The concentration of QYT used in this study was determined based on our preliminary experiment, as well as on the clinical dosage (Ji et al., 2015) that was converted to dosage in mice with minor modification.

**Table 1:** Number of animals for different experimental groups and various parameters.

	NS	NS + QYT	LPS 4 h	LPS 24 h	LPS + QYT	Total
HR, SBP, and DBP	10	10	10	10	10	50
Leukocyte adhesion	6	6	6	6	6	30
Albumin leakage						
MCP-1, GM-CSF, MIP-1α, TNF-α, IL-1α, IL-1β, and IL-6 in plasma and brain tissue homogenate	8	8	8	8	8	40
Cathepsin B activity in plasma and brain tissue homogenate						
Western blot assay						
Evans blue extravasation	6	6	6	6	6	30
Water content	6	6	6	6	6	30
Nissl stain, TUNEL stain, and immunofluorescence	3	3	3	3	3	15
Total	39	39	39	39	39	195

*The animals used for albumin leakage were the same as those for detection of leukocyte adhesion. The animals used for detection of cathepsin B activity in plasma and brain tissue homogenate, and western blot were the same as those for detection of MCP-1, GM-CSF, MIP-1α, TNF-α, IL-1α, IL-1β, and IL-6 in plasma and brain tissue homogenate*.

### Identification of Composition in Rat Plasma After Oral Administration of Qing-Ying-Tang

Two male Wistar rats, weighting around 250 g, received the granule of QYT (15 g/kg body weight) by gavage, and the blood collection was performed at 15 min, 30 min, 1 h, 2 h, 4 h, 6 h, 10 h, and 24 h after administration and frozen at −20°C until analysis. Twenty microliter plasma samples were added into 400 μl methanol. The samples were mixed for 1 min and then centrifuged at 13,000 rpm for 10 min. The supernatant was transferred to Waters Q-TOF/MS system for further analysis. Separation was conducted on a Waters T3 column (2.1 mm × 100 mm, 1.8 μm) at 35°C. The ultraviolet spectra were recorded at 203/254/280/330 nm. The flow rate was 0.3 ml/min. Mass spectrometry was carried out for full scan analysis in the mass range of m/z 50–1,500 in positive and negative ionization mode, respectively. Mode MS^E^: the collision energy of dissociation was set from 20 to 60 eV. Mode MS/MS: the collision energy of dissociation was set at 20 eV.

Acute toxicity studies show that a maximum six times the experimental dose is still safe.

### Assessment of Heart Parameters and Survival Rate

The mice were monitored for heart parameters including heart rate (HR), systolic blood pressure (SBP), and diastolic blood pressure (DBP) at baseline and 2, 4, and 24 h after LPS administration by using an oscillometric device (Softron BP-98E; Softron, Tokyo, Japan). The survival rate of animals was recorded over a period of 48 h.

### Observation of Microcirculation Dynamics

Under anesthesia with 2% pentobarbital sodium (60 mg/kg body weight, i.p.), mice were subjected to intubation *via* the left femoral vein and grinding on left parietal bone using a hand-held drill (STRONG-90; Saeshin, Daegu, Korea) to reveal the cerebral cortical microvasculature. Venules with a diameter of 35–45 μm and a length of 200 μm were chose for study.

To assess adherent leukocytes, rhodamine 6G served as a fluorescence tracer to label leukocytes, which was administrated at 1.5 mg/kg body weight to mice through the femoral vein. Ten min thereafter, the cerebral microcirculation was probed by an upright intravital fluorescent microscope (BX51WT; Olympus, Tokyo, Japan) equipped with a CCD camera (USS-301; Uniq, Santa Clara, United States) using a helium-neon laser beam for illumination. Venular images were acquired under irradiation at wavelength of 543 nm, and used for evaluation of adherent leukocytes, which were identified as cells that remained on the venular walls for more than 10 s (Kurose et al., 1994). The number of adherent leukocytes was scored at 0, 1, 2, 4, and 24 h after LPS infusion and expressed as the number per 200 μm of venule length.

To assess albumin leakage from venules, the mice received FITC-albumin (50 mg/kg) by infusion through femoral vein. Ten min thereafter, a super-sensitive CCD camera (USS-301; Uniq, Santa Clara, United States) was applied to acquire fluorescence signal at excitation wave length of 420–490 nm and emission wave length of 520 nm. The fluorescence intensities of FITC-albumin within the venules (Iv) and outside the venules (Ii) were evaluated by use of Image-Pro Plus 5.0 software, and Ii/Iv served as a measure of albumin leakage. The ratio of albumin leakage at a time point to that of the baseline was designated as the ratio of albumin leakage at that point (Zhang et al., 2016).

### Determination of Cytokines Concentration in Plasma and Brain Tissue Homogenate

At 4 or 24 h after LPS challenge, the mice were killed and blood and brain tissue were gathered. The concentrations of MCP-1, GM-CSF, MIP-1α, TNF-α, IL-1α, IL-1β, and IL-6 in plasma and brain tissue were determined by flow cytometry using a BD cytometric bead array kit (BD Biosciences, San Jose, United States) (Zhao et al., 2012) according to the instruction of manufacture. Briefly, following anticoagulation with heparin (20 unit/ml), the blood was centrifuged at 1,300 g, 4°C for 10 min to separate plasma, in 50 μl of which bead was added in a proportion of 1:1 (v/v), and incubated at room temperature in dark for 1 h. Phycoerthrin-labeled antibody (50 μl) was then added and incubated for 2 h at room temperature followed washing with 1 ml washing buffer. Brain tissue was homogenized, centrifuged, and processed the same way. The mean fluorescence intensity of cytokines was assessed by flow cytometry (FACS Calibur; BD Biosciences, San Jose, United States), and the data were analyzed by the BD cytometric bead array analysis software (Zhang et al., 2014).

### Frozen Sections

Frozen sections were prepared for histology. For this propose, mice under anesthesia were subjected to infusion through the left ventricle with normal saline (NS) followed by 4% paraformaldehyde in 0.1 M PBS for 40 min. Brains were collected and further fixed in 4% paraformaldehyde overnight. After infused in 30% sucrose at 4°C for 2 days, the samples were embedded in TissueTek OCT compound (Miles Inc., IN, United States), frozen in 2-methylbutane cooled in liquid-nitrogen. Coronal brain sections were prepared with a cryostat microtome (CM1900; Leica, Nussloch, Germany) at −20°C. After mounted and air-dried, the brain sections were stored at −20°C (Gu et al., 2018).

### Nissl Staining

Nissl staining was conducted on the sections using cresyl violet acetate as reported (Gu et al., 2018). The result was evaluated with a light microscope (BX512DP70; Olympus, Tokyo, Japan).

### Immunofluorescence Staining

Immunofluorescence staining was undertaken as routine. Specifically, after antigen retrieval and blocking of nonspecific binding, the following primary antibodies were applied to the sections: mouse anti-claudin-5 (1:50), mouse anti-occludin (1:50), rabbit anti-VCAM-1 (1:50), mouse anti-Iba1 (1:400), rabbit anti-CD68 (1:400), rabbit anti-CD18 (1:200), mouse anti-caveolin-1 (1:100), rabbit anti-collagen IV (1:100), and rabbit anti-vWF (1:200). Negative control was performed simultaneously to confirm the specificity of antibodies. After washed by PBS for three times, the specific binding was recognized by the following secondary antibodies: Dylight 488-labeled goat anti-rabbit IgG (1:100, KPL, Gaithersburg, MD, United States) and Dylight 549-labeled goat anti-mouse IgG (1:100, KPL, Gaithersburg, MD, United States). Hoechst 33342 (Molecular Probes) was applied to display the nuclei. The results were evaluated by use of a laser scanning confocal microscope (TCS SP8; Leica, Mannheim, Germany). For quantitative analysis, three sections were chose for each group and five fields were randomly selected in section. Analysis was done using Image -Pro Plus 5.0 software.

### TUNEL Assay

Apoptotic neurons were detected by TUNEL assay using an *in situ* cell death detection kit (Roche, Basel, Switzerland) as per the instruction of the manufacturer. The results were evaluated using a laser scanning confocal microscope (TCS SP8; Leica, Mannheim, Germany).

### Evans Blue Leakage and Brain Water Content

To delve into the BBB breakdown, Evans blue leakage in brain was first determined as previously described (Hu et al., 2009). In brief, mice received Evans blue dye (4% in NS, 3 ml/kg) by injection *via* the right femoral vein. Four h later, the animals were anesthetized with pentobarbital sodium (0.1 g/kg body weight, i.p.) and transcardially perfused with NS. Brains were taken out, homogenized in PBS and centrifuged at 2,000 × *g* for 15 min at 4°C. Then, 0.5 ml of the resultant supernatant was mixed with trichloroacetic acid in a proportion of 1:1 (v/v), incubated overnight and centrifugated at 2,000 × *g* for 15 min at 4°C. The supernatant was subjected to spectrophotometric quantification of leaked Evans blue dye at 620 nm. Brains water content was measured alike as described (Hu et al., 2011). For this, brains were removed from mice under anesthesia, weighed (wet weight) and then dried in an oven at 80°C for 72 h and weighed again (dry weight). The percentage of water content was calculated according the equation [(wet weight − dry weight)/wet weight] × 100% (Huang et al., 2012).

### Determination of Cathepsin B Activity

At 4 or 24 h after LPS challenge, the mice were sacrificed and blood and brain tissue were taken. Cathepsin B activity was determined using an cathepsin B assay kit (Invitrogen Corporation, Camarillo, CA, United States) according to the instruction of the manufacturer. The change in absorbance was measured spectrophotometrically at 460 nm for determination of Cathepsin B activity.

### Western Blot

For western blot analysis, animals in NS and LPS groups were sacrificed at 4 and 24 h, respectively, while animals in NS + QYT and LPS + QYT groups were sacrificed at 24 h. Brains were removed and cerebral cortex were used for preparation of whole cell proteins by RIPA buffer. Protein samples were separated using a 10% Tris-HCl precast gel by electrophoresis (Bio-Rad Laboratories, Hercules, CA, United States) at 80 V for 120–150 min, transferred to polyvinylidene fluoride membranes. Following blocked with 3% skimmed milk in TBST the membranes were incubated overnight at 4°C with primary antibodies against ICAM-1 (1:2000), VCAM-1 (1:2,000), claudin-5 (1:200), occludin (1:1,000), JAM-1(1:1,000) and ZO-1 (1:1,000), VE-cadherin (1:1,000), caveolin-1 (1:1,000), p-caveolin-1 (1:500), MMP-9 (1:1,000), collagen IV (1:1,000), TLR-4 (1:400), Src (1:1,000), p-Src (1:500), NF-κB p65 (1:1,000), NF-κB p-p65 (1:500), NF-κB p50 (1:1,000), and GAPDH (1:4,000). After washed with TBST for three times, specific bindings were recognized with the respective horseradish peroxidase-conjugated secondary antibody (1:5,000, Santa Cruz Biotechnology, Santa Cruz, CA, United States) by incubation at room temperature for 60 min and revealed using enhanced chemiluminescence detection kit (Applygen Technologies, Beijing, China). Band intensities were quantified by densitometry and presented as mean area density using Quantity One image analyzer software (Bio-Rad; Richmond, CA, United States).

### Data Analysis

Data analysis was carried out using one-way ANOVA or two-way ANOVA followed by Bonferroni test and expressed as mean ± SEM with a value of *p* < 0.05 being considered statistically significant.

## Results

### A Total of 10 Compositions of Qing-Ying-Tang Are Found in Rat Plasma

**Figure 1:**
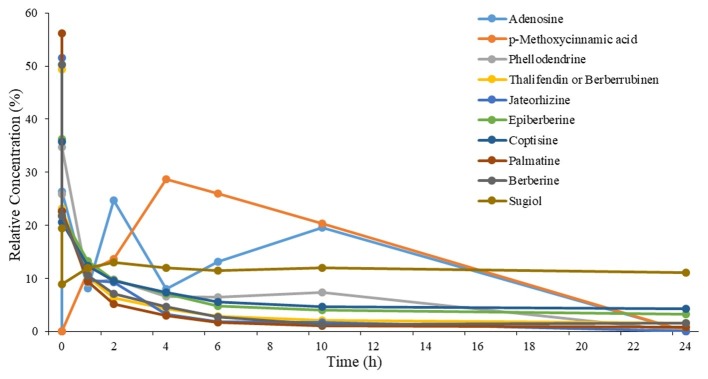
Relative concentration-time profiles of the 10 components in plasma after oral administration of QYT to rats. *n* = 2.

A total of 10 compositions of QYT were revealed in rat plasma following oral administration. At each time point, the relative concentration of components in rat plasma (peak area/Σpeak area) × 100% was presented in Figure 1. Pharmacokinetic parameters, including like Tmax and T1/2, were calculated by the software PKSolveyh. The pharmacokinetic parameters of each compound were summarized in Table 2.

**Table 2:** The pharmacokinetic parameters of 10 absorbed components in rat plasma after administration of the QYT.

Compound	*T*_max_ (h)	*T*_1/2_ (h)
Adenosine	0.5	/
*p*-Methoxycinnamic acid	4	12.09
Phellodendrine	0.5	/
Thalifendin or berberrubinen	0.25	17.40
Jateorhizine	0.25	7.62
Epiberberine	0.25	22.03
Coptisine	0.25	31.74
Palmatine	0.25	11.99
Berberine	0.25	16.48
Sugiol	0.25	227.71^*^

*The absorption rates of alkaloids were relatively fast after the rats were administered with QYT. And the peak time of blood concentrations were within 15 min*.

* *There may be error*.

### Qing-Ying-Tang Post-treatment Relieves the Vital Sign Depravation and Increases the Survival Rate After Lipopolysaccharide Stimulation

**Figure 2:**
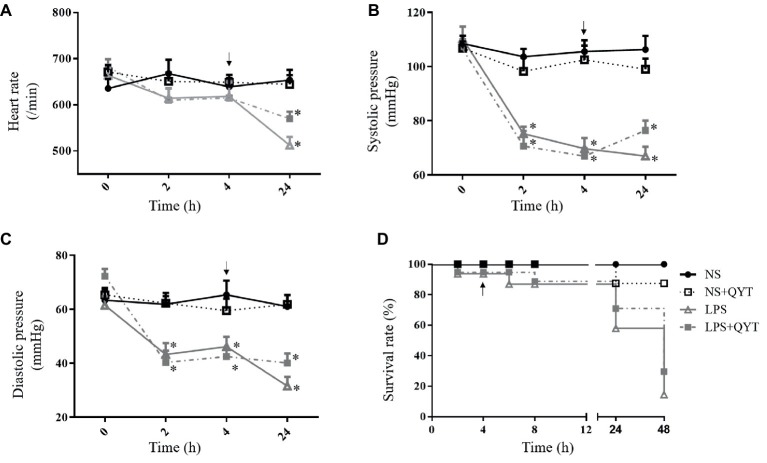
The effect of QYT post-treatment on the physiological parameters in different groups. **(A)** HR. **(B)** SBP. **(C)** DBP. **(D)** Survival rate. Arrows indicate the time when QYT was administrated. Values are mean ± SEM. ^*^p < 0.05 vs. NS group, *n* = 10.

Figures 2A–C present respectively the results of the HR, SBP and DBP of the mice in different groups using a vital parameter monitor recorded at 0, 2, 4, and 24 h after LPS stimulation. As noticed, LPS stimulation resulted in a significant depravation of vital signs manifesting a decrease in HR 24 h after LPS challenge, and in SBP and DBP starting from 2 h after LPS till 24 h, which were all relieved by post-treatment with QYT. Moreover, the survival rate of the animals decreased significantly starting from 24 h after LPS administration, which was improved by QYT (Figure 2D).

### Qing-Ying-Tang Decreases the Leukocytes Adhesion to Brain Venules and Ameliorates the Increased Expression of VCAM-1

**Figure 3:**
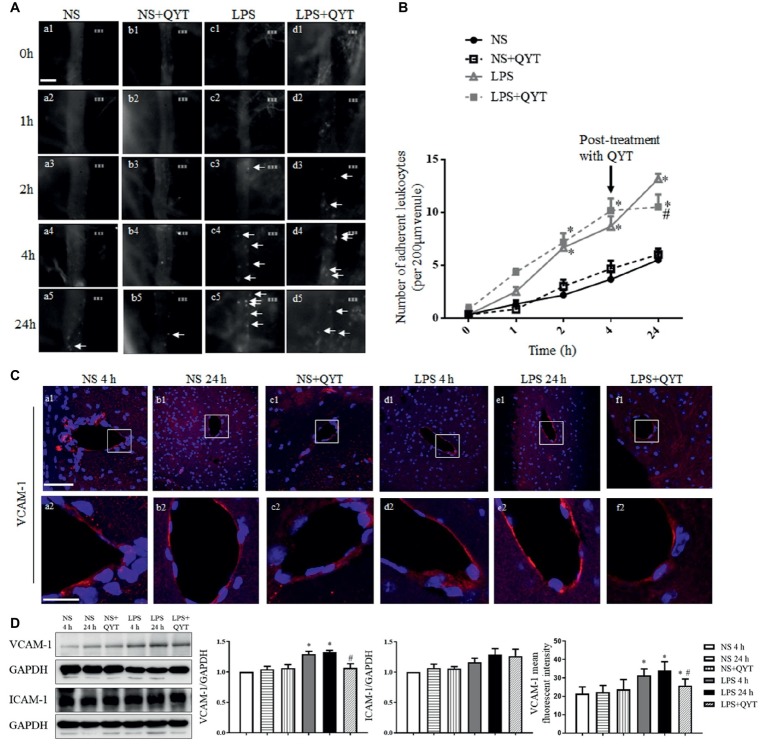
QYT attenuates leukocytes adhesion to cerebral venules and protects the upregulation of VCAM-1 after LPS. **(A)** Representative images of leukocytes adherent to cerebral venules in different groups acquired at different times. Arrows refer to adherent leukocytes. Bar = 50 μm. **(B)** Statistic analysis of the number of leukocytes adhering to cerebral venules in various groups. Values are the mean ± SEM. ^*^p < 0.05 vs. NS group, ^#^*p* < 0.05 vs. LPS group, *n* = 6. **(C)** The representative immunofluorescence images of VCAM-1 in different groups acquired by confocal microscope. Red color denotes VCAM-1 and blue color represents nuclei. The rectangle region in each picture **(a1-f1)** is magnified and shown below **(a2-f2)** correspondingly. Bars = 100 μm in **(a1-f1)**, Bars = 25 μm in **(a2-f2)**. **(D)** Representative western blot bands and quantitative analysis of VCAM-1 and ICAM-1, and statistic result of VCAM-1 fluorescent intensity. All the quantifications were conducted based on the data of four independent experiments with GAPDH as a loading control. Values are the mean ± SEM. ^*^p < 0.05 vs. NS group, ^#^*p* < 0.05 vs. LPS 24 h group, *n* = 4 for western blot, *n* = 3 for immunostaining.

To assess the role of QYT in the brain venule dysfunction after LPS challenge, the leukocyte adhesion to brain venules was examined. Figure 3A shows the images of brain venules which stand for what observed in various conditions at 0, 1, 2, 4, and 24 h after LPS stimulation. Two hours after LPS instillation, the number of leukocytes adhering to venules increased significantly and did not recover at 24 h (Figures 3A,C). Post-treatment with QYT after LPS instillation can significantly reduce the number of leukocyte adhesion at 24 h (Figures 3A,D). A quantification of the number of adhering leukocytes in different groups confirmed the results (Figure 3B). VCAM-1 and ICAM-1 are critical adhesion molecules that recruit leukocytes to inflamed areas. Thus, the immunofluorescence staining of VCAM-1 was conducted, and the expression of VCAM-1 and ICAM-1 was assessed by western blot for mice brain from various groups. As noticed in Figures 3C,D, the expression of VCAM-1 increased in LPS group. This LPS-elevated VCAM-1 expression was relieved by QYT treatment; whereas, no significant difference in ICAM-1 was noticed among groups.

### Qing-Ying-Tang Diminishes the Pro-inflammatory Cytokine Concentration in Mice Brain Tissue After Lipopolysaccharide Challenge

**Figure 4:**
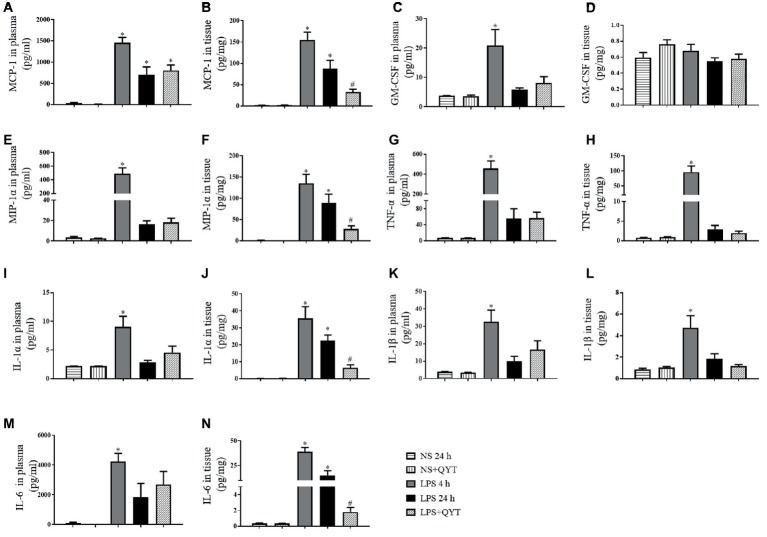
Effect of QYT on the concentration of pro-inflammatory cytokine in mouse brain and plasma after LPS challenge. **(A)** The concentration of MCP-1 in plasma; **(B)** The concentration of MCP-1 in brain tissue. **(C)** The concentration of GM-CSF in plasma; **(D)** The concentration of GM-CSF in brain tissue. **(E)** The concentration of MIP-1α in plasma; **(F)** The concentration of MIP-1α in brain tissue. **(G)** The concentration of TNF-α in plasma; **(H)** The concentration of TNF-α mouse brain tissue. **(I)** The concentration of IL-1α in plasma; **(J)** The concentration of IL-1α in brain tissue. **(K)** The concentration of IL-1β in plasma; **(L)** The concentration of IL-1β in brain tissue. **(M)** The concentration of IL-6 in plasma; **(N)** The concentration of IL-6 in brain tissue. The cytokines were assessed by flow cytometry at 4 and 24 h after LPS stimulation, respectively. Values are the mean ± SEM. ^*^p < 0.05 vs. NS group, ^#^*p* < 0.05 vs. LPS 24 h group, *n* = 8.

To explore the effect of QYT on the inflammatory process elicited by LPS, the concentrations of MCP-1, GM-CSF, MIP-1α, TNF-α, IL-1α, IL-1β, and IL-6 in mice plasma and brain were examined. As shown in Figure 4, all pro-inflammatory factors but GM-CSF in bran tissue increased significantly at 4 h after LPS instillation, and recovered to different extent after 24 h of LPS instillation. Of notice, the LPS-elicited elevation in MCP-1, MIP-1α, IL-6, and IL-1α in brain tissue was decreased significantly by QYT, implying the potential of QYT to attenuate the inflammation process evoked by LPS. On the other hand, no effect of QYT on the level of proinflamatory cytokines in plasma was observed.

### Qing-Ying-Tang Reduces the CD68 and CD18 Positive Cells and Modulates the Lipopolysaccharide-Induced Alternation of Src and NF-κB

**Figure 5:**
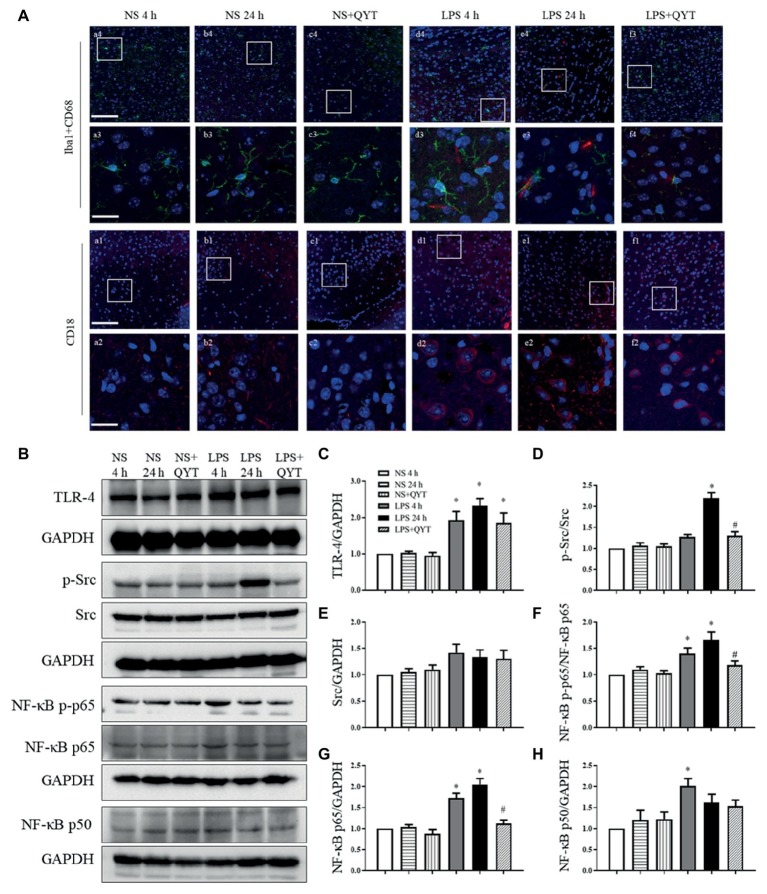
QYT reduces the number of CD68 and CD18 positive cells and modulates the LPS-evoked change in Src and NF-κB. **(A)** Representative immunofluorescence confocal images of Iba1 co-labeling with CD68 and CD18 acquired by confocal microscope in different groups. Red color denotes CD68 **(a1-f1, a2-f2)** and CD18 **(a3-f3, a4-f4)**, green color denotes Iba1 **(a1-f1, a2-f2)** and blue color represents nuclei. The rectangle region in pictures numbered 1 and 3 are enlarged and presented below as picture numbered 2 and 4, correspondingly. Bars = 100 μm in **(a1-f1, a3-f3)**, Bars = 25 μm in **(a2-f2, a4-f4)**. **(B)** Representative western blot bands of TLR-4, p-Src, Src, and NF-κB (p-p65, p65, and p50) in different groups. Shown on the right side are the quantitative analysis of TLR-4 **(C)**, p-Src **(D)**, Src **(E)**, NF-κB [p-p65 **(F)**, p65 **(G)**, p50 **(H)**] in different groups, respectively. All the quantifications were conducted based on the data of four independent experiments with GAPDH as a loading control. Values are the mean ± SEM. ^*^p < 0.05 vs. NS group, ^#^*p* < 0.05 vs. LPS 24 h group, *n* = 4.

CD68 and CD18-positive cells were evaluated in various conditions by immunofluorescence, and the results are displayed in Figure 5A. In comparison with NS (Figures 5Aa,Ab) groups, numerous CD68 and CD18-positive cells were found in LPS 4 h (Figure 5Ad) and LPS 24 h (Figure 5Ae) groups, hinting leukocyte infiltration in brain after LPS challenge. This LPS provoked leukocyte infiltration was relieved considerably by QYT treatment (Figure 5Af). To explore the signaling involved in the role of QYT, the expression of TLR-4 and Src and activation of NF-κB in brain tissue were determined by western blot. As noticed in Figures 5B–H, LPS challenge for 4 and 24 h led to an elevated expression of TLR-4 (Figures 5B,C), expression and phosphorylation of Src (Figures 5B,D,E), and activation of NF-κB (Figures 5B,F–H) in brain tissue. Post-treatment with QYT had no effect on the augmented expression of TLR-4 and p-Src by LPS, while attenuated the increased expression of Src and activation of NF-κB, implying involvement of these signaling in the beneficial effect of QYT.

### Qing-Ying-Tang Relieves the Increase in Cerebral Vascular Permeability After Lipopolysaccharide

**Figure 6:**
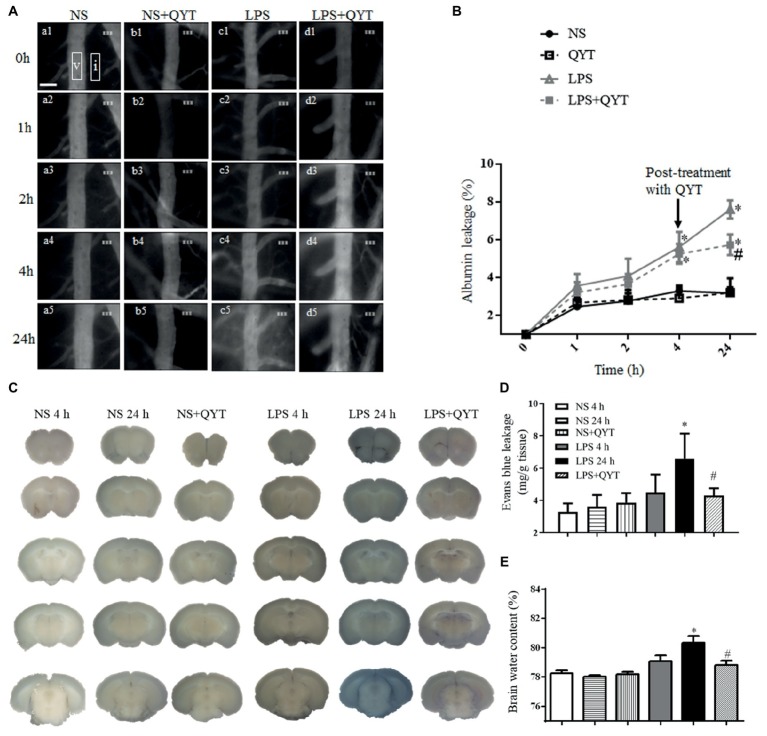
QYT reduces albumin leakage, Evans blue extravasation, and brain water content. **(A)** Pictures representing FITC-albumin leaked from cerebral venules in different groups. Rectangles are the regions on which fluorescence intensity was determined. V, cerebral venule. I, interstitial tissue. Bar = 50 μm. **(B)** Time course of change in the albumin leakage from cerebral venules in various groups. Values are the mean ± SEM. ^*^p < 0.05 vs. NS group, ^#^*p* < 0.05 vs. LPS group, *n* = 6. **(C)** Photos representing Evans blue leakage in the brains in different groups. **(D)** Quantification of Evans blue leakage. **(E)** Brain water content. Values are the mean ± SEM. ^*^p < 0.05 vs. NS group, ^#^*p* < 0.05 vs. LPS 24 h group, *n* = 6.

Cerebral vascular permeability was assessed in various groups by detecting the dynamics of FITC-labeled albumin leakage from cerebral venules. As shown in Figure 6A, fluorescence was observed in the venules and barely outside in NS and NS + QYT groups. In contrast, an obvious fluorescence was visible in perivascular areas in both LPS and LPS + QYT groups 1 h after LPS infusion (Figures 6Ac2,Ad2), which was further augmented in the LPS group at 2, 4, and 24 h (Figures 6Ac3–Ac5). Surprisingly, QYT post-treatment reduced albumin leakage even though microvascular permeability had increased (Figure 6Ad5). These results were further verified by a quantification of dynamics of albumin extravasation (Figure 6B).

Consistent with albumin leakage, Evans blue dye extravasated markedly in the brain of mice in LPS 24 h group compared with those in NS and NS + QYT groups. Post-treatment with QYT decreased the LPS-evoked extravasation of Evans blue dye significantly (Figures 6C,D). Meanwhile, QYT treatment significantly diminished the brain water content as compared with LPS 24 h group (Figure 6E). All in all, these results prove the capacity of QYT to relieve the LPS-provoked increment of BBB permeability.

### Qing-Ying-Tang Alleviates Downregulation of Junction Proteins and Upregulation of Caveolae and Caveolin-1 in Endothelial Cells

**Figure 7:**
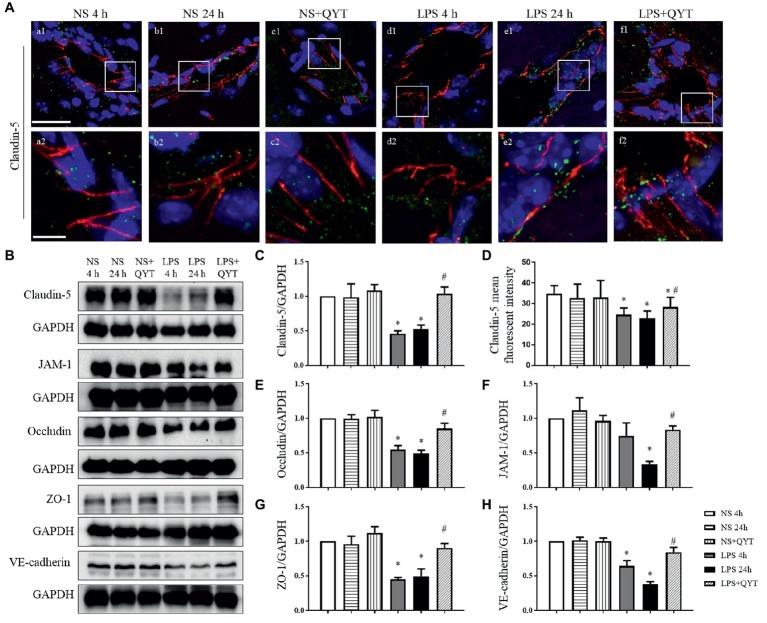
QYT relieves the reduced expression of junction proteins in vascular endothelial cells. **(A)** The immunofluorescence confocal pictures showing claudin-5. Claudin-5 (red) localized at peripheral of the endothelial cells which were marked by VWF (green). Blue color shows nuclei. The rectangle region in each picture numbered 1 is magnified and shown below as pictures numbered 2, correspondingly. Bars = 50 μm in **(a1-f1)**, Bars = 7.5 μm in **(a2-f2)**. **(B)** Representative western blots of claudin-5, occludin, JAM-1, ZO-1 and VE-cadherin in different groups. Shown on the right side is the quantification of claudin-5 **(C)**, occludin **(E)**, JAM-1 **(F)**, ZO-1 **(G)**, VE-cadherin **(H)**, respectively. **(D)** The quantitative analysis of claudin-5 fluorescent intensity. All the quantifications were performed based on the data of four independent experiments and normalized to GAPDH, respectively. Values are the mean ± SEM. ^*^p < 0.05 vs. NS group, ^#^*p* < 0.05 vs. LPS 24 h group, *n* = 4 for western blot, *n* = 3 for immunostaining.

To explore the reason for the amelioration effect of QYT on protecting BBB from breakdown, vascular endothelial TJ and AJ proteins were evaluated by confocal microscopy and western blot. Confocal microscopy showed claudin-5 as continuously intermediate lines between endothelial cells in NS and NS + QYT groups (Figures 7Aa1,Aa2,Ab1,Ab2,Ac1,Ac2). While in LPS 4 h and LPS 24 h groups (Figures 7Ad1,Ad2,Ae1,Ae2), these continuous distributions became interrupted, accompanying with a reduced immune staining, implying a lessened expression of claudin-5 in LPS stimulation groups. Importantly, this lessened expression was ameliorated by post-treatment with QYT (Figures 7D,Af1,Af2). Similar results were obtained alike by western blot (Figures 7B,C). Additionally, western blot found that the expression of other 3 components of TJs including occludin, JAM-1 and ZO-1, and the important component of AJs, VE-cadherin varied among groups in a similar manner (Figures 7B,E–H). These results indicated that QYT attenuated the reduction in TJ and AJ expression induced by LPS, which may at least partly account for the role of QYT in protection of BBB from breakdown.

**Figure 8:**
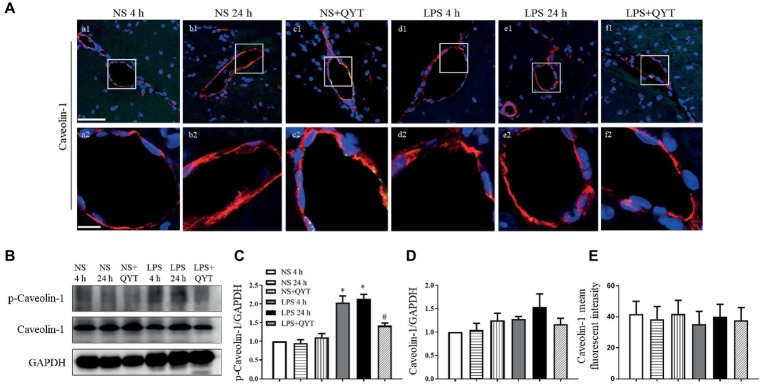
QYT reduces the expression and phosphorylation of caveolin-1. **(A)** Representative immunofluorescence confocal photographs of caveolin-1 (red) in different groups, wherein nuclei are revealed by blue color. The rectangle region in each picture numbered 1 is magnified and shown below as picture numbered 2, correspondingly. Bars = 50 μm in **(a1-f1)**, Bars = 10 μm in **(a2-f2)**. **(B)** Representative western blots of p-caveolin-1 and caveolin-1. Shown on the right side is the quantification of p-caveolin-1/ caveolin-1 **(C)** and caveolin-1/GAPDH **(D)**. **(E)** Quantification of caveolin-1 fluorescent intensity. All the quantifications were conducted based on the data of four independent experiments and normalized to GAPDH, respectively. Values are the mean ± SEM. ^*^p < 0.05 vs. NS group, ^#^*p* < 0.05 vs. LPS 24 h group, *n* = 4 for western blot, *n* = 3 for immunostaining.

Caveolae are structures that mediate endothelial cell permeability by transcellular transport with cav-1 as the main component. In present study, confocal microscopy revealed no obvious change in the expression of cav-1 in brain among groups (Figures 8A,E). However, western blot showed an increased cav-1 phosphorylation in LPS 4 h and LPS 24 h groups (Figures 8B,C), while no difference in the cav-1 expression was observed among groups (Figure 8D). Noticeably, the LPS caused increment in cav-1 phosphorylation was blunted significantly by post-treatment with QYT (Figure 8C). These results suggested that QYT attenuates the BBB breakdown after LPS stimulation by intervention of both inter endothelial and transendothelial pathway.

### Qing-Ying-Tang Relieves the Lipopolysaccharide-Evoked Damage of Collagen IV and Increased Expression of MMP-9 in Brain, Protects Cathepsin B Activation in Plasma and Brain

**Figure 9:**
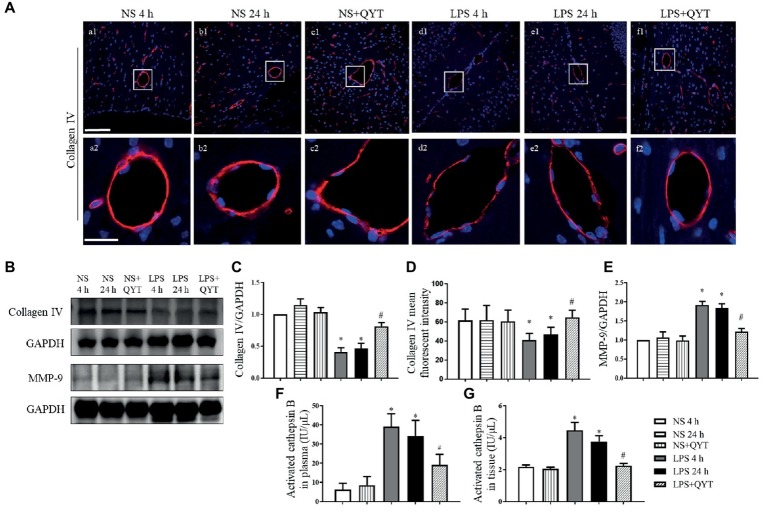
The effect of QYT on LPS-caused alteration in collagen IV, MMP-9, and cathepsin B. **(A)** Representative immunofluorescence confocal photos of collagen IV (red), wherein blue color shows nuclei. The rectangle region in each picture numbered 1 is magnified and presented below as picture numbered 2. Bars = 100 μm in **(a1-f1)**, Bars = 25 μm in **(a2-f2)**. **(B)** Representative western blots of MMP-9 and collagen IV. Shown on the right side is the quantitative analysis of collagen IV **(C)** and MMP-9 **(E)**. Quantifications were conducted based on the data of four independent experiments using GAPDH as a loading control. **(D)** Quantitative analysis of collagen IV fluorescent intensity. **(F,G)** Quantitative analysis of activated cathepsin B in plasma **(F)** and in brain tissue **(G)** in various conditions. Values are the mean ± SEM. ^*^p < 0.05 vs. NS group, ^#^*p* < 0.05 vs. LPS 24 h group, *n* = 6.

Collagen IV examined by confocal microscopy exhibited an continuous distribution around the endothelium in brain in NS and NS + QYT groups, while LPS instillation led to a discontinuity of collagen IV, which was relieved by treatment with QYT (Figures 9A,D). These results were proved by western blot (Figures 9B,C). The expression of MMP-9 was further assessed by western blot and the result showed that the contents of MMP-9 in brain tissue were apparently increased after LPS challenge, while post-treatment with QYT protected the LPS-induced increase in MMP-9 (Figures 9B,E). This result suggested involvement of inhibition of MMP-9 expression in the role of QYT in protecting microvascular basement membrane after LPS challenge. Cathepsin B has been known to cleave type IV collagen (Victor et al., 2011; Shoji et al., 2014; Tong et al., 2015) and participate in the regulation of BBB. The present study observed an increase in cathepsin B activation in plasma as well as in brain tissue of mice exposed to LPS, which was significantly repressed by QYT post-treatment (Figures 9F,G).

### Qing-Ying-Tang Protects Against the Neuronal Damage and Apoptosis Induced by Lipopolysaccharide

**Figure 10:**
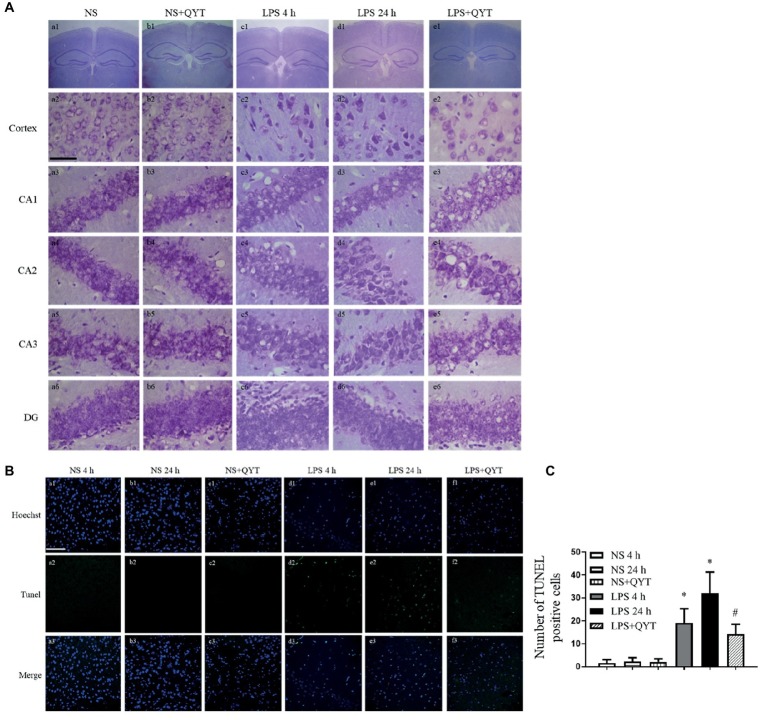
QYT prevents the neuronal injury and apoptosis caused by LPS. **(A)** Brain neocortex and hippocampus sections are processed for Nissl staining. Bar = 50 μm. **(B)** Brain neocortex tissue sections are processed with TUNEL staining. Bar = 100 μm. **(C)** Quantitative analysis of TUNEL positive cells.

The effect of QYT post-treatment on neuron morphology in neocortex and CA1, CA2, CA3, and DG region of hippocampus after LPS stimulation was assessed by Nissl staining (Figure 10). Neurons of NS group (Figures 10Aa,Ab) exhibited normal features in morphology, while those in LPS 4 h and LPS 24 h groups showed a diversity of damages including cell swelling, nuclear contraction, and crush (Figures 10Ac,Ad). Post-treatment with QYT effectively prevented the neuronal damages induced by LPS (Figure 10Ae). TUNEL assay was carried out to assess neuron apoptosis after LPS stimulation, and the result found that a large number of TUNEL-positive cells was present in the cortex region of mice exposed to LPS (Figures 10Bd,Be), whereas TUNEL-positive cells were scarcely visible in NS and NS + QYT groups (Figures 10Ba–Bc). The number of TUNEL-positive cells was decreased in QYT post-treatment groups in comparison with LPS 24 h groups (Figure 10Bf).

## Discussion

Encephalopathy is one of the manifestations of endotoxemia. The present study demonstrates that post-treatment with QYT significantly attenuates the LPS-induced cerebral microcirculation disorders of mice, including decreased leukocyte adhesion and albumin efflux, along with a reduction of cytokines in brain tissue. Meanwhile, QYT treatment elevated HR and increased SBP and DBP, improved the hypodynamic status and survival rate of mice.

Cerebral microvascular exudation caused by LPS plays a pivotal role in pathogenesis of sepsis-associated encephalopathy which causes high brain pressure and edema, accounting for the occurrence of disturbed consciousness of sepsis (Coisne and Engelhardt, 2011; Tauber et al., 2017). Despite clinically commonly used hormone-supportive therapy, dehydration and antihypertensive approaches, the cerebral edema and disturbance of consciousness in sepsis have not been completely resolved. The present study demonstrates the beneficial role of QYT in LPS-induced cerebral microvascular hyperpermeability, as evidenced by the decrease in brain Evans blue exudation and brain water content, suggesting QYT as a potential therapy to counteract this threat.

Cerebral microvascular exudation is a consequence of breakdown of BBB, to which endothelium and basement membrane are major contributors (Abbott et al., 2010). In present study, LPS challenge impaired endothelial cells junctions by downregulation of tight junction proteins, increased caveolae by activation of caveolin-1, and damage on basement membrane by enhanced expression of MMP-9 and cathepsin B, which collectively led to BBB breakdown. Of notice, QYT treatment relieved all the changes after LPS, thus protected BBB from disintegrity. LPS-caused BBB breakdown is mediated through a range of signaling, including activation of multiple protein-tyrosine kinases, NF-κB activation and nuclear translocation, oxidative stress, and release of pro inflammatory cytokines, all initiating with binding of LPS to TLR-4 and taking place successively and coordinately (Gong et al., 2008; Kawai and Akira, 2011). It is at present unknown exactly which signaling (s) be implicated in the observed effect of QYT. We have previously reported that catalpol, the active ingredient of Rehmannia glutinosa contained in QYT formula, can reduce LPS-induced mesenteric microvascular permeability and hemorrhage by regulating TLR-4 and Src signaling pathways (Zhang et al., 2016). DLA (Wang et al., 2009) and salvianolic acid B (Lin et al., 2013; Pan et al., 2015), the active constituents of Salvia, inhibits the degradation of I-κB, nuclear translocation of NF-κB and activation of Src kinase, inhibits the expression and phosphorylation of Cav-1 and degradation of ZO-1 and therefore protects from mesenteric and pulmonary microcirculation disorders in rat. However, the present study did not reveal the protective effect of QYT on Src activation, nor on TLR-4 expression, thus excluded the involvement of TLR-4 expression and Src signaling in the beneficial role of QYT in microvascular hyperpermeability after LPS. The reason for ineffectiveness of catalpol, DLA, and salvianolic acid B in inhibiting Src activation and TLR-4 expression in the present case is unknown. As to the mechanism for the protective effect of QYT on LPS- caused BBB breakdown, what is clear is that QYT prevented the increased activation of NF-κB after LPS. It is likely that it is the blocking the of NF-κB signaling by QYT that reduced the level of proinflammatory cytokines, protected the downregulation of junctions proteins, and upregulation of MMP-9 and adhesion molecules, thus improved cerebral microcirculation and protected BBB from breakdown after LPS.

QYT consists of nine different components, each of them contains numerous bioactive ingredients. The present study focused on the effect of QYT as a whole on the LPS-induced cerebral vascular hyperpermeability and its underlying mechanism. Much more work is required to elucidate the role of any individual ingredient in QYT in the effect observed.

## Conclusion

This study demonstrated that post-treatment with QYT attenuated LPS-induced cerebral microcirculatory disorder, inhibiting leukocyte adhesion to microvessels, protecting microvascular hyperpermeability as evidenced by the decreased albumin exudation and brain edema, which is correlated with reduced proinflammatory cytokines level and increased expression of junctions proteins.

## Data Availability Statement

All datasets generated for this study are included in the article/supplementary material.

## Author Contributions

H-MW undertook the research, analyzed the data, and wrote the manuscript. PH performed western blotting and analyzed the data of western blotting and immunostaining. QL contributed to the animal experiments. L-LY designed and performed pharmacokinetics and toxicity studies. KS contributed to the measurement of MCP-1, GM-CSF, MIP-1α, TNF-α, IL-1α, IL-1β, and IL-6 in plasma and brain tissue homogenate and the determination of HR, SBP, and DBP. L-LY contributed to the Nissl stain and immunofluorescence. C-SP contributed to the western blotting. Y-YL, C-SW, X-HW, and B-HH contributed to the other experiments. J-YF and PH revised the manuscript. J-YH designed and funded the research, interpreted the data, and finally approved the submission of this manuscript. All authors read and agreed with the manuscript.

### Conflict of Interest

The authors declare that the research was conducted in the absence of any commercial or financial relationships that could be construed as a potential conflict of interest.
